# Reduced Bacterial Biodiversity Is Associated with Increased Allergy

**DOI:** 10.1289/ehp.120-a304

**Published:** 2012-08-01

**Authors:** Sharon Levy

**Affiliations:** Sharon Levy, based in Humboldt County, CA, has covered ecology, evolution, and environmental science since 1993. She is the author of the book *Once and Future Giants: What Ice Age Extinctions Tell Us about the Fate of Earth’s Largest Animals*.

Early humans coevolved with an array of microbes and parasites that modern city dwellers no longer encounter. A growing body of evidence suggests that reduced contact with these ancient microbial partners may be helping to fuel an epidemic of inflammatory diseases such as asthma, allergies, multiple sclerosis, type 1 diabetes, and ulcerative colitis—all of which are on the rise in urban populations.^1^^,^^2^ Now ecology and evolutionary biology professor Ilkka Hanski and his colleagues at the University of Helsinki have observed a link between the environments people inhabit, the diversity of microbes residing on their skin, and their susceptibility to allergic reactions.^3^

In a study of teenagers living in urban and rural environments in eastern Finland, Hanski’s team used molecular analysis to show that allergic children hosted a less diverse array of bacteria on their skin compared with healthy counterparts. In addition, children living in homes with a greater diversity of native flowers in the yard had a more varied array of microbes on their skin and a lower risk of allergy. The expression of interleukin-10, a key anti-inflammatory cytokine, was positively correlated with abundance of one particular genus, *Acinetobacter*, on skin.^3^

The diversity of plant and animal communities shapes the diversity of microbial communities—and a number of environmental microbes can interact with human immune systems in beneficial ways.^3^ “If we lose our connection to environmental microbiota, there may be adverse consequences to our immunologic tolerance,” explains Hanski.

The findings fit with the “old friends” hypothesis, which holds that organisms that coexisted with early hominids came to shape human immune responses.^1^ Examples include helminths, bacteria common in soils and in the human gut, and viruses such as hepatitis A. “These organisms had to be tolerated, because attacking them would lead to pointless tissue-damaging inflammation,” says Graham Rook, emeritus professor of medical microbiology at the Centre for Clinical Microbiology at University College London.

Some organisms that coexist with humans may produce molecules that activate regulatory T lymphocytes, which block inflammation. For instance, in experimental animal models, an array of chronic inflammatory diseases, including type 1 diabetes, colitis, arthritis, and asthma, can be blocked by infection with helminth parasites.^4^ A study of thousands of children living in Austria, South Germany, and Switzerland found that exposure to a greater diversity of bacteria and fungi was associated with less allergies and asthma in rural children.^5^ Rook notes that the observed relationship between abundant *Acinetobacter* and increased expression of interleukin-10 suggests that Hanski’s team has documented a genuine case of immunoregulation by environmental bacteria.

**Figure:**
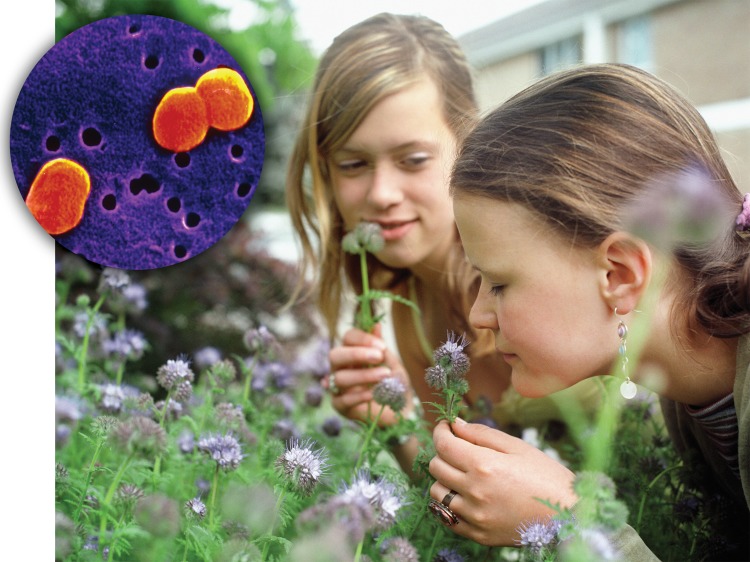
A greater diversity of native flowering plants in the yard appears linked to exposure to Acinetobacter species of bacteria (inset), which may promote allergy resistance. © Mika/Corbis; Inset: CNRI / Photo Researchers, Inc.

Although the current study sampled microbiota on the skin of children’s forearms, Rook suspects that any biological effect of *Acinetobacter* and other organisms occurs when they are inhaled and encounter immune cells in the airways. “We do not know the relative importance of contact via the skin and via the airways,” he says, “but physiologically the airways seem more likely.” If these organisms can be confirmed as true immunoregulatory agonists, Rook says it suggests that “old friends” could be added back to the modern environment by rather simple means such as potted plants colonized with beneficial organisms.

For Hanski, the notion of distributing microbes artificially is not enough. “It is essential to retain contact with natural habitats,” he says, “especially in the case of young children. Our findings highlight the importance of green space in urban areas, and of opportunities for urban children to spend some time in the countryside.”

Hanski and his colleagues plan to extend their study to a contrasting population in Russian Karelia—where rates of asthma, allergies, and type 1 diabetes are much lower than those in nearby eastern Finland.^1^ Although the people of eastern Finland and Russian Karelia are genetically similar, the Russian side of the border is less affluent and has lower levels of hygiene.^6^ Hanski hopes to quantify the differences in microbial diversity in the two regions to clarify the role of “old friends” in protecting against allergy.
